# Donor Oath: Respect to the Mortal Teacher to Learn Ethics and Humanitarian Values of Anatomy

**DOI:** 10.7759/cureus.22941

**Published:** 2022-03-07

**Authors:** Sathvika SV, Yuvaraj Maria Francis, Balaji Karunakaran, Gunapriya Raghunath, Kumaresan M, Zareena Begum, Rajalakshmi Subramanian

**Affiliations:** 1 Anatomy, Saveetha Institute of Medical and Technical Sciences, Chennai, IND; 2 Anatomy, Tagore Medical College and Hospital, Chennai, IND

**Keywords:** anatomy, donor, learning, humanitarian, ethics

## Abstract

The basic framework of the term "respect" is equality recognition. Students and teachers in academic institutions can have varying definitions of respect. Respect in the learning environment is critical for student success. This study aims to look into how students' perceptions of the human body in the anatomy lab and their respect for them can be used to promote ethical obligations, humanitarian principles, and their interrelationships. It is a descriptive, questionnaire-based study with 20 questions and a consent form that involves 250 students in the first year of MBBS. Using Microsoft Excel 2019, the data were collected, tabulated, and interpreted using descriptive statistics. The donor oath is a way to emphasize that the human body in front of them was once alive. The donor oath establishes the groundwork for future efforts to adapt existing frameworks for ethical decision-making and humanitarian values.

## Introduction

William Osler said: "A good physician treats the disease. A great physician treats the patient who has the disease" [1]. A medical professional should be directed by a golden rule: "do unto others as you would have them do unto you and your family," because nothing outperforms helping a patient and receiving their sincere gratitude [2]. Physicians and medical students should have sensitivity, compassion, empathy, and respect for the patient’s dignity, privacy, and confidentiality. The four basic elements of the doctor-patient relationship are trust, knowledge, regard, and loyalty [3]. However, there is currently a loss of credibility between patients' families and doctors. With the advancement of technology, there is a growing expectation for better results. When a successful result is not obtained, there are assaults on physicians and other healthcare personnel by patients' friends and relatives. Any negative outcome is thought to be the result of carelessness [4]. The term "donor" would have a more positive meaning than "cadaver" or "corpse." This simple change in the terminology can also modify the students' attitudes and empathy towards the deceased individual.

To improve the doctor-patient relationship, the National Medical Council of India has introduced bioethics in the medical curriculum to train medical undergraduates from the first day of their entry into medical college [5]. Learning bioethics is a significant way to learn medical professionalism, and it must be initiated in the dissection hall itself. The dissection of bodies will help the medical students gain knowledge through observation, identification, and palpation of various structures in the body. The spatial and tactile nature of the body may help students differentiate the normal and anatomical variations that occur in the human body [6]. The three-dimensional view of the different structures observed in the human body can be well appreciated in the anatomy lab and can be used for educational and scientific research purposes. Though there are diverse methods to learn anatomy, the traditional use of human bodies in teaching still plays a major role in the medical curriculum [7].

Human dissection was the dominant means of teaching and learning anatomy in Alexandria, and it was also followed by Herophilus of Chalcedon and his younger contemporary, Erasistratus of Ceos, who became the first ancient Greek physician to perform systematic dissection of deceased humans [8]. Biological systems are complex and extensive. The deeper a student reads and discusses, the more the student learns. Human bodies are required to study the gross anatomy of human structures. A deceased human is the first thing a medical student learns about knowledge, conduct, and altruistic behavior. As a result, these deceased individuals need to be held in the highest respect and regarded as medical students' first patients. In spite of various mixed emotions towards desecration, dismemberment, dehumanization, etc., medical students should have moral respect for the bodies while handling them [9-11]. Then respect may develop towards patients in the future. The deceased individual is possessed by a person who chose to donate his or her body in order to give back to society. Students must acknowledge these donors' kindness and compassion in having given their bodies as passive mentors in the medical college.

Selflessness, which is understood from a body donation, is a basic professional competence. It emphasizes the holistic aspect of medicine and is intended to cultivate in students the morals that will direct them to become expert and compassionate doctors. Taking a donor oath is now an important component of bioethics in order to experience the altruistic behavior of a donor. The aim of this study is to determine the significance and perceptions of Phase I MBBS students towards the donor oath.

## Materials and methods

This study is an observational survey that involved 250 (160 females and 90 males) phase I MBBS students and was conducted in the Department of Anatomy, Saveetha Medical College and Hospital. Three sessions were designed to promulgate humanistic attitudes in first-year undergraduate medical students. The first session was "Donor as the First Teacher." The session was conducted in the Department of Anatomy during the foundation course, and its aim was to document the affective domain of students about "how to respect the deceased human and treat the bodies." The second session was a donor oath performed in front of the human body, and sensitized advice was given by the faculties. The third session was done to identify the perceptions and attitudes towards the donor oath and its significance among the Phase I MBBS students.

The questionnaire was prepared and standardized by peer faculties, then shared with students through Telegram, WhatsApp, and e-mail. There were nine questions to evaluate the perceptions and attitudes of students towards handling human bodies. The students were asked to respond according to the 6 point Likert scale. The 10th question analyzed the students’ feelings towards the donor, and finally, the students were asked to upload their creative thoughts on the topic "Donor as a First Teacher." The responses obtained in the study were statistically analyzed using descriptive statistics in SPSS software (IBM Corp., Armonk, NY).

## Results

**Figure 1:**
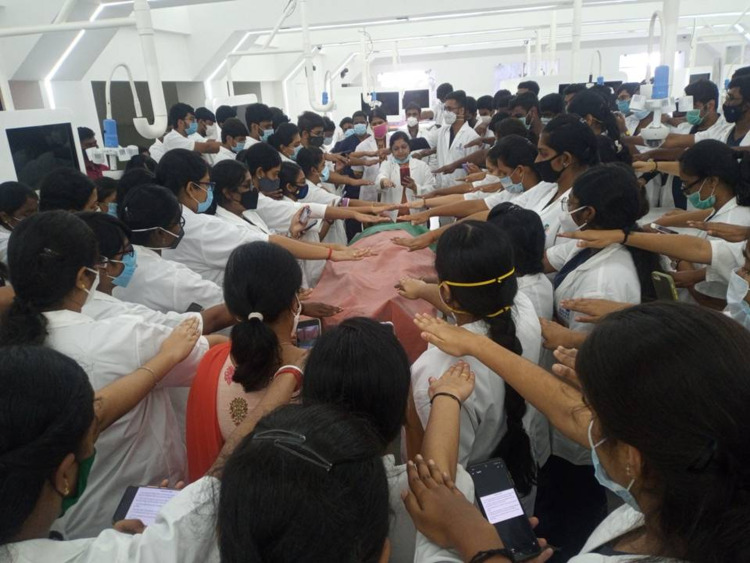
The donor oath taken by phase I MBBS students

**Figure 2:**
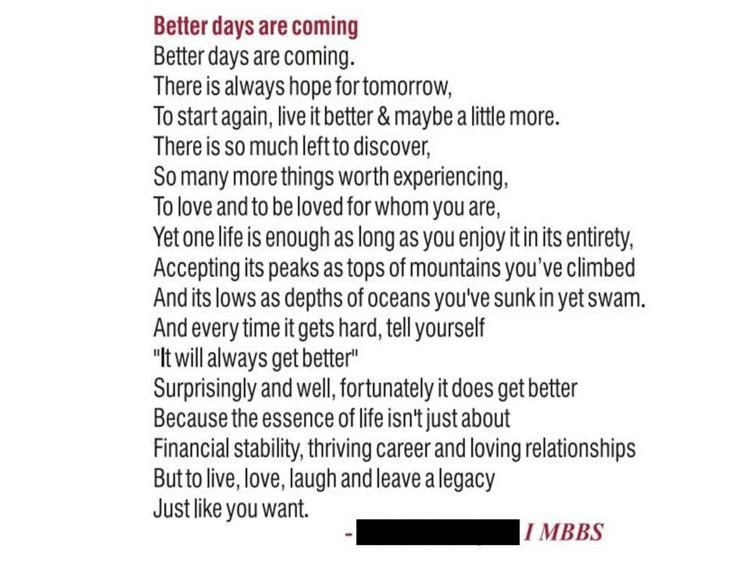
An English poem penned by a phase I MBBS student

**Figure 3:**
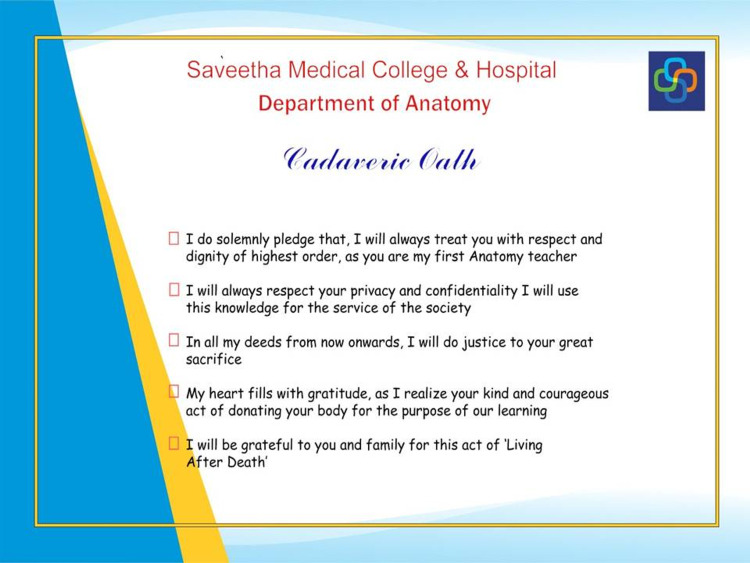
Donor oath

A total of 250 phase I undergraduate medical students (160 females and 90 males) completed the questionnaire-based survey towards the perceptions and attitudes of donor oath and its significance. The response rate is 100% since the questionnaire was completed by all the students during their AETCOM module (Attitude, Ethics, and Communication Module) in the classroom (Figures 1-2). Around 238 (95.2%) students strongly agreed that a cadaveric oath was necessary to learn and understand anatomy. According to 242 (96.8%) students, the implementation of the donor oath in the foundation course was essential to understanding and orienting themselves to medical education. 240 (96%) students agreed that the introduction of bioethics in the medical curriculum would improve the doctor-patient relationship. 212 (84.8%) students responded that bodies in the anatomy lab should be handled in a respectful and dignified manner. Around 248 (99.2%) students agreed that they should be thankful to the family members for donating the body. Around 88.5% of students agreed that the donor oath had helped to overcome the emotional shock at the initial stage of exposure and handling the bodies. According to 97.6% of students, dissection was the best way to learn anatomy instead of emerging new techniques like virtual human dissection tables, 3D molded models, and computer-aided teaching. Of 250 students, 218 have shared their creative ideas about handling and respecting deceased humans in different ways, such as poems and paintings. The responses obtained in the study are shown in Table 1 and Figure 3.

**Table 1:** Phase I MBBS students’ responses to the Likert scale feedback towards donor oath and its significance. The responses were: strongly disagree (1), disagree (2), slightly disagree (3), slightly agree (4), agree (5), strongly agree (6).

Questions	Responses (%, mean ± SD)
Do you feel that a donor oath is necessary to learn anatomy?	88%
Do you feel that introduction of donor oath in the foundation course is necessary?	4.97 ± 0.81
Do you feel that the introduction of bioethics into the medical curriculum will improve the doctor-patient relationship?	5.06 ± 0.13
Do you feel that donors should be handled in a respectful and dignified manner?	5.21 ± 0.27
Do you feel that a donor oath helps in overcoming emotional shock at initial exposure to the donor?	4.81 ± 0.49
Do you feel thankful for family members for donating the body of their relative for academics, workshops, and research purposes?	5.35 ± 0.41
Do you feel that donor teaching techniques can be replaced by plastic models and computer-aided teaching in the near future?	4.91 ± 0.36
Do you think that the donor oath has a role in developing empathy and acting as a silent mentor?	5.16 ± 0.19
Do you think that dissection enhances the students to improve creative thinking?	4.83 ± 0.37
Do you feel that donor teaching is more understandable in comparison with others methods of teaching?	5.07 ± 0.19

## Discussion

The aim of the present study was to investigate the perceptions of phase I MBBS towards the donor oath and its significance in learning anatomy. Anatomy is considered the basis of clinical medicine. Anatomy can be learned and taught in diverse ways, but the traditional teaching through dissection and disarticulated specimens is highly valuable. In spite of the availability of many kinds of teaching and learning methods in the medical field, students and faculties are more attracted towards using donors in their teaching, mainly due to their realistic nature, 3D orientation, appreciation, and handling of various structures of the human body, which could help in differentiating normal anatomy from pathological lesions [12-16]. Students are not inclined towards other teaching methods when compared with using donors' bodies in the anatomy laboratory. According to Zehra et al., medical undergraduates who took part in dissection performed well in both theory and practical exams [17]. In this study, around 96.7% of medical undergraduate students were satisfied with teaching using donors' bodies in comparison with other teaching methods. The results of this study were well correlated with the observations of Alhassan and Majeed and Izunya et al. [18,19]. The formulation of values needed for a good doctor-patient relationship, such as respect, responsibility, and appreciation, can never be achieved by the use of modern technology-based tools. To maintain professional ethics from the beginning, it is desired that medical undergraduates take a donor oath before initiating human body dissection. In this study, 95.2% of phase I MBBS strongly agreed that the donor oath played a significant role in learning anatomy, and its introduction into the foundation course is a must as per the suggestions of the National Medical Council to improve ethics and imbibe the strong doctor-patient relationship among medical students [20]. The results of the study were correlated with the results of research conducted among students in Taiwan by Chiou et al. [21].

To maintain the principles of medical ethics such as respect for autonomy, beneficence, non-maleficence, and justice, the donor oath was performed among our phase I medical students, as it is followed in most medical institutes around the globe (Figure 1).

A donor oath is a way for students to emphasize the truth that the body in front of them was once alive [22]. Furthermore, the donor oath allows students to better understand the eventual destination of life and realize the true value of human life. Remarkably, the existing literature from Asian countries focuses on a specific phrase in this regard, "donor oath," so even elsewhere it is a suitable addition to the Hippocratic Oath. The medical students would give respect to their silent mentors as they begin their professional careers [23].

Medical students undergo various kinds of emotions like stress, anxiety, fear, and lack of concentration before seeing and handling the human body. These emotional experiences can be mitigated through the donor oath. In this study, around 87.6% of students experienced that the donor oath had reduced many emotional problems while handling the human body in the initial phase of learning. Apart from knowledge rendering, the dissection hall is a suitable place to inculcate professionalism, humanities, etc., in young doctors.

According to Kaba et al., students and faculty who dissect and handle the specimens appreciate the family members of the donor [24]. In this study, about 95% of phase I MBBS students strongly agreed that family members must be thanked for their noble gesture of donating their relative’s body for academic and research purposes. In our institute, students invite the family members of the body donors for thanksgiving. Oklahoma College of Medicine also conducts thanksgiving for the donor’s family members to maintain humanity and dignity [25,26].

Finally, in this study, most of the students expressed their creativity by penning beautiful poems, drawings, etc. The creative response obtained from the students is shown in Figure 2. The observation of this study correlated well with the response of a study conducted by Anne et al. at Manipal Medical School [27].

In recent decades, the development of web-based teaching and learning has reduced the humanitarian values of the medical profession. The main objective of the donor oath is to develop empathy and compassion, and clinically excellent healthcare professionals who will treat each other and every future patient with the respect and dignity that they deserve. Those who gain practical experience will gain responsiveness and will always recognize the human within the patient.

## Conclusions

The implementation of bioethics in the medical curriculum as per the suggestion of the National Medical Council will help the MBBS students to learn and understand the ethics and humanitarian values for their future medical profession. It will develop values like empathy, respect, and care for patients and their family members. A donor oath, carried out on the first day of entry into the dissection hall, will inculcate respect for the silent mentor and aid in building a good doctor-patient relationship. Medical students must remember that life is precious before and after death, and they should never forget the human body as their first anatomy teacher.
